# Systemic immune response and virus persistence after foot-and-mouth disease virus infection of naïve cattle and cattle vaccinated with a homologous adenovirus-vectored vaccine

**DOI:** 10.1186/s12917-016-0838-x

**Published:** 2016-09-15

**Authors:** Michael Eschbaumer, Carolina Stenfeldt, Steven I. Rekant, Juan M. Pacheco, Ethan J. Hartwig, George R. Smoliga, Mary A. Kenney, William T. Golde, Luis L. Rodriguez, Jonathan Arzt

**Affiliations:** 1 United States Department of Agriculture (USDA), Plum Island Animal Disease Center (PIADC), Foreign Animal Disease Research Unit (FADRU), Agricultural Research Service (ARS), P.O. Box 848, Greenport, NY 11944 USA; 2 Oak Ridge Institute for Science and Education, PIADC Research Participation Program, Oak Ridge, TN USA

**Keywords:** FMDV, Vaccination, Persistence, Carrier, Flow cytometry, Lymphopenia, Interferon, ELISA, ELISPOT

## Abstract

**Background:**

In order to investigate host factors associated with the establishment of persistent foot-and-mouth disease virus (FMDV) infection, the systemic response to vaccination and challenge was studied in 47 steers. Eighteen steers that had received a recombinant FMDV A vaccine 2 weeks earlier and 29 non-vaccinated steers were challenged by intra-nasopharyngeal deposition of FMDV A24. For up to 35 days after challenge, host factors including complete blood counts with T lymphocyte subsets, type I/III interferon (IFN) activity, neutralizing and total FMDV-specific antibody titers in serum, as well as antibody-secreting cells (in 6 non-vaccinated animals) were characterized in the context of viral infection dynamics.

**Results:**

Vaccination generally induced a strong antibody response. There was a transient peak of FMDV-specific serum IgM in non-vaccinated animals after challenge, while IgM levels in vaccinated animals did not increase further. Both groups had a lasting increase of specific IgG and neutralizing antibody after challenge.

Substantial systemic IFN activity in non-vaccinated animals coincided with viremia, and no IFN or viremia was detected in vaccinated animals. After challenge, circulating lymphocytes decreased in non-vaccinated animals, coincident with viremia, IFN activity, and clinical disease, whereas lymphocyte and monocyte counts in vaccinated animals were unaffected by vaccination but transiently increased after challenge. The CD4^+^/CD8^+^ T cell ratio in non-vaccinated animals increased during acute infection, driven by an absolute decrease of CD8^+^ cells.

**Conclusions:**

The incidence of FMDV persistence was 61.5 % in non-vaccinated and 54.5 % in vaccinated animals. Overall, the systemic factors examined were not associated with the FMDV carrier/non-carrier divergence; however, significant differences were identified between responses of non-vaccinated and vaccinated cattle.

**Electronic supplementary material:**

The online version of this article (doi:10.1186/s12917-016-0838-x) contains supplementary material, which is available to authorized users.

## Background

Foot-and-mouth disease virus (FMDV; family *Picornaviridae*; genus *Aphthovirus*) causes a highly contagious, acute disease of cloven-hoofed animals, with fever, lameness, and vesicular lesions of the feet, tongue, muzzle, and teats reviewed in [1–3]. Foot-and-mouth disease (FMD) is a difficult and expensive disease to control and eradicate due to its wide host range, low minimum infectious dose, rapid rate of replication, high level of viral shedding, and multiple modes of transmission [1, 3]. The situation is further complicated by an important subclinical divergence that occurs after acute infection of ruminants: some animals remain subclinically infected for up to 3 years (“FMDV carriers”) ([2], reviewed in [4, 5]), while others completely clear the virus within 1 to 2 weeks (“non-carriers”). The definition of an FMDV carrier established by the World Organisation for Animal Health (OIE) is an animal from which infectious FMDV can be recovered at greater than 28 days after infection [6].

The bovine nasopharynx [7–12] and regional lymph nodes [13] have been identified as sites of this persistence, but it is poorly understood how FMDV evades clearance by the host immune response at these sites [14]. It is also unknown whether there are pre-existent factors or patterns in the virus-host interaction during and after acute infection that can be used to predict or influence the ultimate outcome of virus clearance versus persistence.

Type I and type III interferons (IFN) are important parts of the early innate immune response to viral infection and are often crucial in controlling or eliminating infection (reviewed in [15]). All cells in the body are responsive to type I IFNs, whereas the type III IFN receptor is mostly restricted to gastrointestinal and airway epithelia [16]. Several reports have demonstrated strong IFN activity during FMDV infection in cattle using an Mx/CAT reporter system which does not differentiate between IFN type I and type III [17–22]. Using this method, type I/III IFN activity has been found in circulating plasmacytoid dendritic cells (pDCs) [18, 23] and in tissues at sites of virus replication [22]. However, it is unclear how much of the systemically detected IFN originates within the vasculature as opposed to from sites of infection in tissues.

In pigs, FMDV infection leads to lymphopenia and immune suppression, manifested as a significant loss of circulating T cells [24, 25]. Significant lymphopenia during acute FMDV infection of cattle has been described [20, 26], but other studies have reported that no changes occur in total circulating leukocytes or relative lymphocyte subpopulations [27, 28]. One report concluded that the T-cell response to mitogen and non-FMDV antigens was not impaired during acute FMDV infection, but no FMDV-specific T-cell responses were detected [28].

In earlier experiments, the depletion of CD4^+^ cells in vivo significantly reduced neutralizing antibody titers and delayed class switching in cattle vaccinated with inactivated FMDV [29]. However, in non-vaccinated cattle, CD4^+^ depletion before FMDV infection had no effect on clinical signs, the induction of neutralizing antibodies, or the acute clearance of virus from the circulation [27]. These and other studies have concluded that the antigenic structure of the FMDV capsid, the high local antigen concentration, and the strong cytokine response during acute infection likely are key factors in the efficient induction of T cell-independent antibody responses [29, 30].

CD8^+^ cytotoxic T lymphocytes (CTLs) from vaccinated pigs are capable of selectively killing FMDV-infected cells in vitro [31], and infection of pigs with FMDV also leads to a clear CTL response [32]. However, in cattle, partial depletion of CD8^+^ cells did not affect the resolution of acute FMDV infection [27]. Given that the acute phase of an FMDV infection is concluded before a significant adaptive CTL response can be mounted [28], it is likely that the control of the infection is mediated by a T-cell-independent neutralizing antibody response and type I/III interferon signaling. Overall, the role of bovine antigen-specific T cells in FMDV infection remains unresolved, and it is unclear how FMDV evades the CTL response during persistent infection.

The kinetics of circulating FMDV-specific antibody-secreting cells in the context of antibody levels and neutralizing activity have not yet been examined. FMDV infection generally elicits a rapid, strong, and lasting antibody response. Coincident with the first detection of antibody there is a rapid clearance of virus from the circulation and a more gradual reduction of virus shedding. Although circulating antibodies are generally believed to be the primary mediators of immunity after infection or vaccination [3], it is well known that vaccines prevent viremia and generalized disease, but not primary local infection, e.g., in the pharynx. Several studies have reported shedding of infectious FMDV in nasal, oral, or oropharyngeal fluids of vaccinated animals following virus exposure [10, 33–37], which is consistent with primary infection of the upper respiratory or gastrointestinal tracts. Virus replication in the nasopharyngeal mucosa of vaccinated animals in the present study has been demonstrated in a separate publication [38]. The occurrence of persistent, asymptomatic FMDV infection in vaccinated cattle [9, 10, 34, 36, 37, 39, 40] provides further unequivocal evidence that vaccination does not prevent primary infection.

Similarly, antibodies are ineffective in clearing virus from the pharynx of carrier ruminants [4], and substantial antibody levels – in serum as well as in secretions-have been reported in animals that remained persistently infected with FMDV [21, 41–43]. In vaccinated animals, protection against challenge is correlated with neutralizing antibody in circulation, but low antibody levels can also be protective [35, 44, 45] and animals with high neutralizing titers can develop disease after challenge [37].

The overarching goal of the present study was to elucidate systemic host factors associated with the response to FMDV in cattle during early and late stages of infection and to categorize these responses in the context of vaccination status and carrier-state divergence. For this purpose, serological and hematological parameters as well as lymphocyte sub-populations were investigated in vaccinated and non-vaccinated cattle from the day of FMDV vaccination and/or infection to the persistent/recovered phase. Trends associated with acute disease, vaccination, and the development of the FMDV-carrier state were examined in the context of clinical, virological, and serological data collected from the same animals.

## Methods

### Animals

Forty-seven Holstein steers (6 to 8 months, ~200 kg) were obtained from an experimental livestock provider (Thomas D. Morris Inc., Reisterstown, MD, USA) accredited by the Association for Assessment and Accreditation of Laboratory Animal Care International and registered with the United States Department of Agriculture (USDA). The animals were housed together in a BSL-3-Ag animal facility from the time of arrival until euthanasia, and were given an acclimation period of 2 weeks before the start of the experiment. The health status of all animals was assessed daily throughout the study period. Based on daily clinical assessments, analgesics and anti-inflammatory drugs (flunixin meglumine, 1.1–2.2 mg/kg; butorphanol tartrate, 0.1 mg/kg) were administered to mitigate pain associated with severe clinical FMD as needed. Steers were sedated with xylazine (intramuscular, 0.22 mg/kg) for inoculations and clinical exams; after the procedure, the sedation was reversed with tolazoline (intravenous, 2 mg/kg).

### Vaccination

Two weeks before infection, eighteen of the 47 steers were immunized using a recently licensed recombinant FMD serotype A vaccine (USDA product code 1FM.1R0; manufactured by Antelope Valley Bios, Lincoln, NE, USA). This vaccine contains the P1-2A and 3Cpro coding regions from FMDV A24 Cruzeiro within a replication-deficient human adenovirus serotype 5 vector [46]. The steers were intramuscularly injected with the product release dose in a 2 mL total volume containing commercially available adjuvant (product #7010101, VaxLiant, Lincoln, NE, USA).

### Challenge infection

All animals were clinically evaluated and sampled prior to inoculation, to ensure their FMDV-free status and the absence of elevated systemic type I/III IFN levels that could interfere with initial FMDV replication. On day 0 of the experiment, all animals were inoculated with 10^5^ infectious doses (titrated in bovine tongue epithelium) [6] of FMDV A_24_ Cruzeiro [47] in 2 mL of minimum essential medium (MEM) with 25 mM HEPES by intranasopharyngeal (INP) deposition [38, 48]. The successful deposition of virus was confirmed in all animals by collection of nasal and oral fluids after removal of the inoculation catheter (data not shown).

### Clinical evaluation

From the day of challenge until 10 days post inoculation (dpi), clinical scores were recorded on a scale from 0 to 5 accounting for presence of FMD vesicles on each foot or anywhere on the head (oral cavity or nasal epithelia) [12]. Clinical examinations with sedation were performed daily in non-vaccinated animals and every other day in vaccinated animals, either throughout the first 10 dpi, or until the animal had reached full clinical score. Rectal body temperatures were taken every day for the entire duration of the experiment.

### Euthanasia and tissue collection

**Fig. 1:**
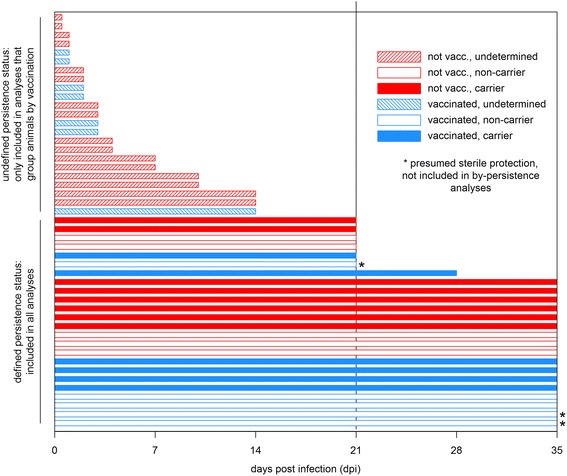
Overview of the experimental timeline. Each bar represents one animal, with the length of the bar corresponding to the time the animal remained in the experiment after challenge infection (21 dpi, the revised cut-off by which FMDV persistence status could be determined, is marked by a *vertical line*). Non-vaccinated animals are shown in *red*, vaccinated animals in *blue*. *Solid-colored bars* are FMDV carriers, *unfilled bars* are non-carriers. *Striped bars* are used when the persistence status of an animal could not be determined because it was euthanized before 21 dpi. Asterisks mark the three animals without convincing evidence of infection (see Results section for details)

A subset of study animals were euthanized at predetermined time points during acute and post-acute phases of infection (0 to 14 dpi) for detailed tissue-based pathogenesis studies which have been presented in separate publications [12, 38]. All samples and data collected before necropsy, however, are included herein. Specifically, four non-vaccinated animals were euthanized on 1 dpi, and two each on 2, 3, 4, 7, 10, and 14 dpi. Among vaccinated animals, two animals were euthanized on each of 1, 2, and 3 dpi, and one on 14 dpi (see Fig. 1). Sodium pentobarbital (intravenous, 86 mg/kg) was used for all euthanasias.

The OIE defines FMDV carriers as animals in which the virus persists for more than 28 days after infection [6]. For the animals in this study, however, it was found that the persistence status could be reliably determined by 21 dpi (see Results section for details).

Since the FMDV carrier status was not determined for animals euthanized earlier than 21 dpi, these animals were excluded from graphical and statistical analyses that discriminated animals by persistence status; however, these animals were included in all analyses that did not require their persistence status to be defined. Twenty-four out of 47 animals (13 non-vaccinated and 11 vaccinated) were kept alive beyond 21 dpi, which was determined to be the threshold at which it was possible to consistently conclude from oropharyngeal fluid samples whether an animal had cleared the infection or entered the FMDV carrier state. With the exception of three vaccinated animals in which infection could not be confirmed (see Results section for details), all animals that were kept alive to 21 dpi or longer were included in all analyses.

### Blood and probang samples

Blood was collected from the jugular vein on the day of vaccination (immediately before), on days 4 and 7 post-vaccination (dpv), on the day of challenge (immediately before), then daily for the first ten dpi and afterwards weekly until 35 dpi. Samples were collected in BD Vacutainer® tubes containing either K_2_EDTA for hematology, heparin for PBMC separation, or serum-separator gel. Starting on 7 dpi (21 dpv) in vaccinated animals and 14 dpi in non-vaccinated animals, oropharyngeal fluids (OPF) were collected by probang cup [49] two times per week. Probang cup contents were mixed with an equal volume of cold MEM with 25 mM HEPES immediately after collection and then kept on ice. Upon arrival in the laboratory, serum tubes were centrifuged for harvesting (10 min at 1000 × g and 4 °C), and OPF samples were immediately processed as described previously [10], including treatment with 1,1,2-trichlorofluoroethane (TTE) to reactivate antibody-bound virus [50].

The PBMC preparation, surface marker staining, and flow cytometric data collection have been described previously [51]. Briefly, for separation of PBMCs, 18 mL of fresh heparinized blood were diluted in Dulbecco’s phosphate-buffered saline (PBS; Life Technologies, Carlsbad, CA, USA), underlaid with Histopaque® 1083 (Sigma-Aldrich, St. Louis, MO, USA), and centrifuged. Harvested PBMCs were washed twice with PBS, counted, and resuspended at a concentration of 10^7^ cells/mL in either fetal bovine serum (FBS; GE Healthcare Life Sciences, Logan, UT, USA) with 10 % (v/v) dimethyl sulfoxide (Sigma-Aldrich) for freezing (for flow cytometry) or in ELISPOT media (RPMI-1640 with antibiotics, 0.1 mM non-essential amino acids, 2 mM L-glutamine, 10 mM HEPES, 1 mM sodium pyruvate [all Life Technologies], and 10 % FBS) for immediate use. Frozen cells were stored at −70 °C for no longer than thirty days before flow cytometry analysis.

### FMDV RNA detection and virus isolation

Real-time RT-PCR and virus isolation for FMDV detection in serum and OPF were performed as previously described [52]. Briefly, FMDV viral RNA was quantified by real-time RT-PCR targeting the 3D region of the FMDV genome [53] with forward and reverse primers adapted from Rasmussen et al. [54]. Samples with cycle threshold (C_t_) values lower than 45 were considered positive. Serial 10-fold dilutions of in vitro synthesized FMDV RNA of known concentration were used to convert C_t_ values to FMDV RNA genome copy numbers (GCN) per mL of sample. After the conversion, the cut-off C_t_ value corresponded to a detection limit of 1.57 log_10_ FMDV GCN/mL.

### Type I/III IFN bioassay

Type I/III IFN activity in serum was quantified using the Mx/CAT reporter assay as previously described [22]. Briefly, serum samples collected during the first 10 days after challenge were incubated for 24 h with recombinant Madin-Darby bovine kidney cells that express chloramphenicol acetyltransferase (CAT) under the control of an Mx1 promoter [17]. CAT expression in the cells was measured with a commercially available ELISA kit (Roche Diagnostics, Indianapolis, IN, USA), and IFN levels in unknown samples were derived from a standard curve of serial dilutions of recombinant human interferon-α 2a with known potency (PBL Assay Science, Piscataway, NJ, USA) that was run in parallel. Results are reported as international units (IU) of IFN per mL of serum. The Mx/CAT assay does not distinguish between type I and type III interferon [20].

### Hematology

For each whole blood sample from all animals, a complete blood count (CBC) was performed on the same day with a Hemavet 950FS veterinary hematology system (Drew Scientific, Waterbury, CT, USA), following the manufacturer’s instructions. High and low values were flagged by the analyzer based on factory-set normal limits. Among the blood parameters reported by the analyzer, only the total white blood cell (WBC) count and its principal components are reported here (in 1000 s [K] of cells per μL of blood). The WBC count is the sum of five subpopulations, with neutrophils, lymphocytes, and monocytes together comprising over 90 % of all circulating white blood cells [55]. The bovine reference ranges are 600 to 4000 neutrophils, 2500 to 7500 lymphocytes, and 0 to 900 monocytes per μL of blood [56].

### Flow cytometry

PBMCs from all animals were evaluated by flow cytometry. Cells were thawed in a water bath at 37 °C, slowly diluted in warm RPMI-1640 media with 10 % FBS, and washed twice with PBS [57]. All samples were stained in duplicate; first with an amine-reactive dye (LIVE/DEAD® Fixable Yellow; Life Technologies), then with monoclonal antibodies against bovine CD3 (MM1A, IgG_1_, Washington State University, Pullman, WA, USA), the δ chain of the γδ T cell receptor (GB21A, IgG_2b_, Washington State University), CD4 (CC8, IgG_2a_, conjugated with FITC, Bio-Rad, Hercules, CA, USA), and CD8 (CC63, IgG_2a_, Alexa Fluor® 647, Bio-Rad), and finally with polyclonal goat antibodies against murine IgG_1_(allophycocyanin-cyanine7; SouthernBiotech, Birmingham, AL, USA) and IgG_2b_ (R-phycoerythrin; SouthernBiotech). Compensation controls and fluorescence-minus-one controls were included for each antibody/dye combination. After each staining step, cells were washed twice in cold FACS buffer (PBS with 0.3 % [v/v] bovine serum albumin fraction V [Life Technologies] and 0.1 % [w/v] sodium azide).

Stained cells were analyzed in a three-laser LSR II flow cytometer (BD). Initially, events were gated based on forward and side scatter, equal pulse height/area ratio (for single-cell selection), as well as live/dead staining behavior, with low dye uptake considered indicative of membrane integrity and cell viability [58]. The boundaries of a morphological lymphocyte gate (defined by forward and side scatter) were established by backgating from CD3. Among all live cells in that gate, T lymphocytes were then identified by CD3^+^ staining. At least 10000 CD3^+^ cells were evaluated per sample. CD3^+^ γδTCR^−^cells were presumed to be αβ T lymphocytes, and were examined for CD4 and CD8 expression. All surface marker gating was done automatically with “snap to” interval gates in single-parameter histograms in BD FACSDiva 8.

The flow cytometer only measures the relative quantities (percent abundance) of T lymphocyte subsets but does not provide absolute cell counts. Because percent abundance can be misleading when assessing the change in a population of cells over different experimental conditions [59], the flow cytometry and hematology data were combined [60] to obtain absolute counts of the CD4^+^ and CD8^+^ αβ T lymphocyte subpopulations. The absolute number of cells in each subpopulation was calculated based on the complete blood count obtained with the Hemavet analyzer as described by Riondato et al. [61]. Briefly, the total lymphocyte counts per mL of peripheral blood were assigned to the morphological lymphocyte gate on the flow cytometer. Absolute numbers for the subpopulations were then obtained by serially applying the percentage-of-parent values to this total count, beginning with the CD3 gate. Absolute counts are reported as number of cells per μL of blood.

### Humoral immunity

All animals that survived for at least 21 days after challenge were included in the serological analyses; animals that were euthanized at earlier time points were not included.

### Serum neutralization test (SNT)

FMDV-neutralizing antibody titers were determined for serum samples taken at − 14 and − 7 dpi (vaccinated animals only; 0 and 7 dpv, respectively), and at 0, 7, 14, 21, 28, and 35 dpi for all animals. Sera were heat inactivated for 30 min at 56 °C and used in a microtiter neutralization assay. Serial fourfold dilutions of serum (in MEM with 25 mM HEPES) on 96-well plates (from an initial dilution of 1/8 down to 1/32768) were incubated with 100 50 % tissue culture infective doses (TCID_50_) of FMDV A24 Cruzeiro for 1 h at 37 °C and 5 % CO_2_. Freshly trypsinized LFBK-αVβ6 cells [62, 63] were resuspended in MEM with 25 mM HEPES, 4 × 10^4^ cells/well were added to the plates, and the plates were incubated for another 72 h at 37 °C and 5 % CO_2_. After microscopic evaluation of cell monolayers, the plates were treated with crystal violet dissolved in tissue fixative (HistoChoice®; AMRESCO, Solon, OH, USA), then washed and air-dried before cytopathic effect was again evaluated visually. Titers were calculated as the reciprocal of the highest dilution of serum that fully neutralized the virus in 50 % of replicate wells.

### FMDV-specific antibody ELISAs

Serum samples were collected on days − 14, −10, −7 (vaccinated animals only), 0 to 10, 14, and 21 after challenge infection and were used without prior heat inactivation. An indirect double antibody sandwich ELISA was developed for the detection of FMDV-specific IgM and IgG in serum. Optimal concentrations of reagents were determined by checkerboard titration. Each reagent was added at a volume of 100 μl per well, except where indicated. All incubations were at 37 °C for an hour, shaking, except where indicated. All washes were performed four times with PBST, 300 μl per well. All dilutions were performed in blocking buffer (BB, 10 % normal horse serum in PBST) except where indicated. Immulon 2HB plates (Thermo Scientific) were coated with anti-FMDV-A polyclonal rabbit serum (Pirbright Institute, Pirbright, United Kingdom) at a dilution of 1/1000 in fresh carbonate/bicarbonate buffer (0.05 M, pH 9.6, Sigma-Aldrich) by incubation overnight at 4 °C. After washing, plates were incubated with 200 μl per well of BB. After emptying plates, they were incubated with positive and negative (made by BEI inactivation of mock-and virus-infected cells) antigen preparations that were added to negative and positive columns, respectively. After washing, serum samples were added at a dilution of 1/100 for IgM detection, 100 μL each on negative- and positive-antigen coated wells. The same was true for IgG detection, but at a dilution of 1/500. In each plate, standard positive and negative sera were included. The standard negative serum was obtained from animals that were FMDV-antibody free. Standard positive sera were chosen during the initial phase of the development of the assays because of their high titer and high maximum absorbance value. After incubation and washing, the bound bovine antibodies were detected using sheep anti-bovine IgM or sheep anti-bovine IgG heavy chain directly conjugated to horseradish peroxidase (A10-101P or A10-118P, respectively; Bethyl Laboratories, Inc., Montgomery, Texas). After incubation and washing, ELISA was completed by addition of substrate solution (SureBlue peroxidase substrate, Kirkegaard & Perry Laboratories [KPL], Gaithersburg, MD, USA) and stopped after 10 min at room temperature by addition of 50 μl/well of stop solution (BlueStop; KPL). Absorbance was measured with an ELx808 microplate reader (BioTek, Winooski, VT, USA) using a 630-nm filter. For each sample, a net OD was calculated by subtracting the reading of the negative-antigen well from the positive-antigen well. For each plate, the net ODs of test samples were then divided by the net OD of the positive control sample on the same plate. Identical aliquots of the same positive control were used for all plates, and results are reported as fractions of the net OD of the positive control (nFPC). To further correct for non-specific reactivity, the nFPC value of the sample taken on the day of first exposure to FMDV (either vaccination or challenge) was subtracted from the nFPC values of all subsequent samples of the same animal.

### FMDV-specific B-cell ELISPOT

FMDV-specific antibody-secreting-cell counts from six non-vaccinated animals that survived until 35 dpi were obtained by ELISPOT. Filter plates (EMD Millipore) were coated with monoclonal antibodies against bovine IgM (IL-A30; 1/1000), IgG_1_ (IL-A60; 1/500) or IgG_2_(IL-A74; 1/25) (International Livestock Research Institute, Nairobi, Kenya) diluted in fresh carbonate/bicarbonate buffer (0.05 M, pH 9.6, Sigma-Aldrich), incubated overnight at 4 °C, and washed and blocked with ELISPOT media (supplemented RPMI-1640 with 12 % horse serum). Fresh PBMCs were serially diluted in ELISPOT media, and 5 × 10^5^, 2.5 × 10^5^ and 1.25 × 10^5^ cells from each sample were seeded in duplicate wells on the same plate, together with a media-only control. After overnight incubation at 37 °C with 5 % CO_2_ and thorough washing with PBS with 0.1 % polysorbate (TWEEN®) 20 (PBST) in an automated plate washer, biotinylated FMDV A24 Cruzeiro antigen (1/500 in PBST) was added to the plates. After a 1-h incubation at room temperature and further washing, HRP-conjugated neutravidin was added at a dilution of 1/1000 (in PBST), and the plates were incubated for 1 h at room temperature and washed again. Captured FMDV antigen was visualized with TrueBlue peroxidase substrate (KPL) and spots were counted with an ImmunoSpot Analyzer (Cellular Technology Limited, Shaker Heights, OH, USA). Spot counts were normalized to the total input of PBMCs, and the IgM count as well as the combined IgG_1_ and IgG_2_ counts were used for comparison with the FMDV antibody ELISA results.

### Statistical analysis of hematology and flow cytometry data

For data analysis, animals were grouped by vaccination or persistence status. Twenty-four animals remained for at least 3 weeks after challenge and were classified as FMDV carriers or non-carriers based on virus isolation results from TTE-treated probang samples. Data were graphed and analyzed with Excel (Microsoft, Redmond, WA, USA) and the R statistical environment [64], particularly the *ggplot2* package [65]. Group means are generally annotated with their 95 % confidence intervals (CI95) to facilitate visual comparisons between groups. For group sizes of 3 or fewer, or where between-group comparisons are not meaningful, standard deviations are shown instead.

The hematology and flow cytometry data were analyzed in R with linear mixed-effects models as implemented in *lme4* [66], using the *car*, *phia*, and *lsmeans* packages for post-hoc analyses of specific linear combinations of factor levels. Two models were built for each outcome variable (white blood cells, neutrophils, lymphocytes, monocytes for hematology, and CD3^+^, CD3^+^γδTCR^−^CD4^+^, CD3^+^γδTCR^−^CD8^+^ for flow cytometry), one with only vaccination status, time, and their interaction term as fixed effects, and another that additionally included persistence status and all interactions between the main effects. This dual approach was chosen because information on persistence status was only available for half of the animals in the study. Animal ID was included as a random effect in all models. For flow cytometry outcome variables, intercepts and slopes were allowed to vary between animals (random intercept and slope), whereas hematology models only had random intercepts.

Where an initial ANOVA (Type II Wald chi-square tests) found significant interactions between status (by vaccination or persistence) and time after infection, pairwise contrasts for the levels of the status factor (vaccination or persistence) were evaluated for each level of the time factor – i.e., difference between vaccinated/non-vaccinated or persistent/non-persistent for each day. Changes in an outcome variable over time were evaluated with custom contrasts (e.g., dpi1 - dpi0) for each level of a status factor; similarly, the effect of time alone was evaluated by averaging across both levels of the status factor and applying the custom contrasts. *P*-values from linear hypothesis tests on the models are reported approximately; any *p*-value <0.05 was considered significant.

For the association between viremia and type I/III IFN activity in serum, the test statistic was calculated using Pearson’s product moment correlation coefficient. The asymptotic 95 % confidence interval is based on Fisher’s Z transform.

The difference between absolute CD4^+^ and CD8^+^ αβ T cell counts in non-vaccinated animals before and after challenge (0 vs. 5 dpi) was evaluated with a paired *t*-test. The variance was estimated separately for both groups and the Satterthwaite approximation to the degrees of freedom was used. Exact *p*-values are reported for the *t*-test.

## Results

### FMDV carrier/non-carrier divergence

The overarching goal of this work was to investigate systemic trends associated with 2 categorical factors: vaccination status at the start of the experiment and FMDV carrier status determined at the end of the study. Animals are defined and stratified as “carrier”, “non-carrier”, or “undetermined” for the entire study based on their final status at the end of the experiment (Fig. 1).

The OIE defines FMDV carriers as animals in which the virus persists for more than 28 days after infection [6]. In the present study, however, all probang samples from non-carriers were virus-negative by 21 dpi and remained negative, whereas all animals that were virus-positive in their 21-dpi probangs remained virus-positive until 28 dpi and beyond; there was no change in viral shedding in probangs in any animal between 21 dpi and the end of the experiment. Thus, for the purposes of this study, FMDV persistence was defined by sustained detection of infectious FMDV in probang samples until at least 21 dpi, or until the day of necropsy, whichever was later. Using this definition, 8 out of 13 non-vaccinated animals (61.5 %) which had survived until or past 21 dpi were determined to be persistently infected carriers, whereas 5 out of 13 non-vaccinated animals (38.5 %) had successfully cleared the infection. Of 11 vaccinated animals, 6 were carriers (54.5 %) and 5 non-carriers (45.5 %). Animals that were euthanized before 21 dpi were assigned an “undetermined” persistence status.

The vaccinated non-carrier group was further segregated into two distinct categories based on the observation that three animals lacked convincing virological evidence of infection. All vaccinated non-carrier animals were similar in that there was no detectable viral RNA or infectious virus in OPF, nor was any infectious virus found in tissue samples taken at necropsy. However, two out of the five vaccinated non-carriers had FMDV RNA in nasopharyngeal tissues at 21 or 35 dpi, indicating previous infection, while the other three did not [12]. Additionally, the three tissue-RNA-negative cattle did not seroconvert against FMDV non-structural proteins, while all other vaccinated and non-vaccinated non-carriers did (not shown). Taken together, this was considered an indication of possible sterile protection, and on this basis, the animals were treated separately and not included with the other non-carriers except for the total count. Specifically, these animals were included in all analyses that stratify animals by vaccination status but were excluded from all analyses that stratify animals by persistence status.

### Clinical disease, viremia, and type I/III IFN response

**Fig. 2:**
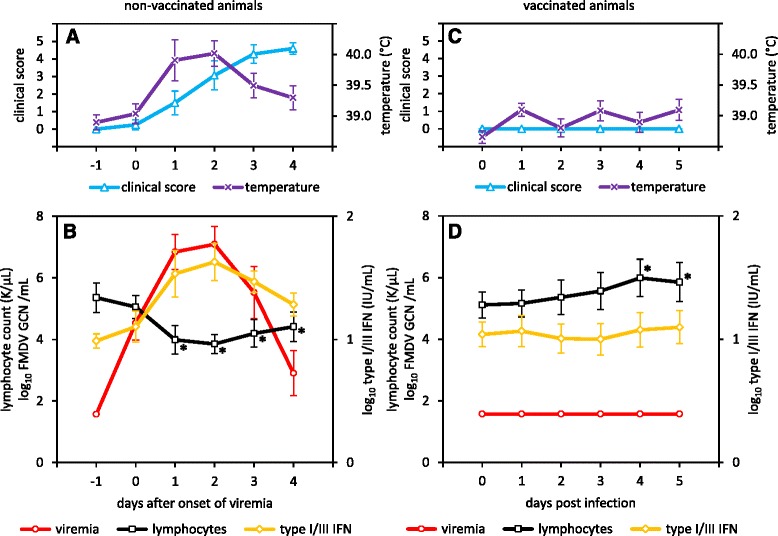
Lymphopenia during acute FMDV infection. **a**/**b** non-vaccinated animals, **c**/**d** vaccinated animals. Mean lymphocyte counts (*black* squares), viral load (*red* circles), and type I/III IFN response (*gold* diamonds) in peripheral blood (**b**/**d**), as well as rectal temperature (*purple* crosses) and cumulative clinical score (*blue* triangles) (**a**/**c**) are shown with 95 % confidence intervals. The group means for non-vaccinated animals were calculated after individual timescales had been adjusted so that the onset of viremia coincided for all animals (0: first day of viremia). Asterisks mark days on which mean lymphocyte counts were significantly different from the initial value in the fitted model. The limit of detection for the FMDV real-time RT-PCR is 1.6 log_10_ FMDV GCN/mL

All non-vaccinated steers developed moderate to severe clinical FMD after virus exposure (Fig. 2a). Characteristic lesions were seen in and around the mouth, including vesicles on the tongue and dental pad as well as on the skin around nostrils and lips. Foot lesions, including vesicles in the interdigital clefts and on coronary bands were often accompanied by moderate lameness. Vesicular lesions first appeared between 2 and 6 dpi (generally on the first day after the onset of detectable viremia) and resolved within approximately 10 days. None of the vaccinated steers developed clinical FMD after challenge (Fig. 2b).

Viremia (defined by presence of viral RNA in serum) was detected in all non-vaccinated steers after virus challenge. The earliest detection of FMDV RNA occurred between 1 and 5 dpi (mean time to initial detection: 3 days), with levels again declining below assay detection limits after four to six days of detection. The peak of mean viremia (6.7 log_10_ FMDV GCN/mL) across all non-vaccinated animals occurred on 5 dpi. Viremia and type I/III IFN activity in serum were significantly correlated (*r* = 0.53 [0.37; 0.66], *p* < 0.01), and peak viremia coincided with peak type I/III IFN activity (Fig. 2a). No difference was seen in clinical disease, the magnitude of the IFN response, or the level of viremia between the groups of animals that subsequently diverged into carriers and non-carriers. FMDV RNA was not detected in sera of any of the vaccinated steers at any time, and there was no detectable type I/III IFN activity in the serum of vaccinated animals after challenge (Fig. 2b). The Mx/CAT assay does not distinguish between type I and type III interferon [20]; hence it was not determined whether the observed interferon response was mediated by type I or type III interferon or both.

### Hematology: vaccinated vs. non-vaccinated animals

**Fig. 3:**
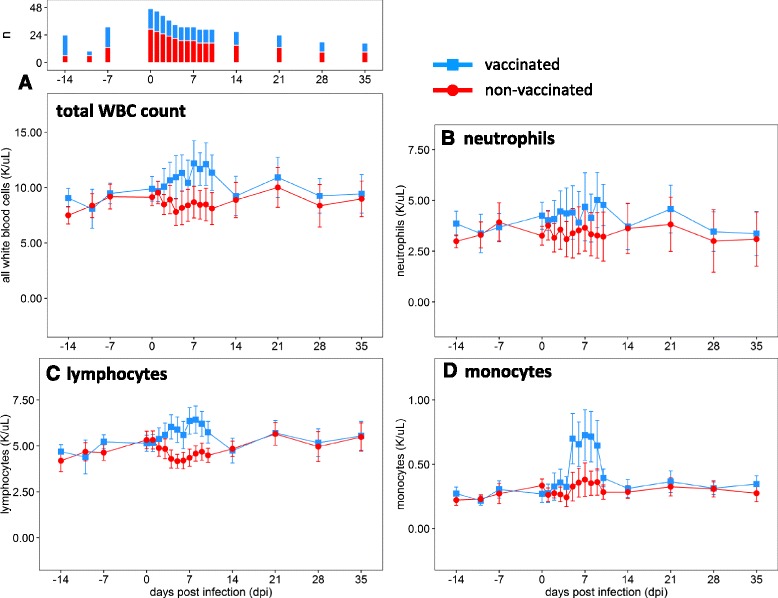
White blood cell subpopulations in vaccinated and non-vaccinated steers. **a** Total WBC count. **b** Neutrophils. **c** Lymphocytes. **d** Monocytes. Animals were assigned to groups based on their FMDV vaccination status, without regard to their persistence status. Vaccinated animals have *blue lines* and *squares*, and non-vaccinated animals have *red lines* and *circles*. The plots show the group means with 95 % confidence intervals. The upper part of panel **a** shows the number of animals (n) that contributed data for each time point; this number is the same for all panels. All 47 animals contribute data to the 0-dpi group means; as animals are being euthanized beginning on 1 dpi, the size of each group decreases

No hematological changes were observed after vaccination, and the two groups (vaccinated and non-vaccinated) did not differ in any cell population in the complete blood count prior to challenge (Fig. 3). All further comparisons are made relative to the day of challenge. After challenge, the mean total WBC count decreased in non-vaccinated animals, while it increased in vaccinated animals. Neither change was significant within the groups, but the difference between non-vaccinated animals and vaccinated animals was significant between 4 and 10 dpi (*p* <0.05) (Fig. 3a). Lymphocyte counts diverged starting at 4 dpi, with vaccinated steers having significantly higher counts than non-vaccinated steers (*p* <0.05) (Fig. 3c). This separation was maintained throughout the early phase of the experiment (until 9 dpi). Within the vaccinated population, mean lymphocyte counts were significantly increased over pre-challenge levels (0 dpi) on 4 and 5 dpi and again from 7 to 9 dpi (*p* <0.05). Lymphocyte counts were significantly decreased in non-vaccinated animals from 4 to 8 and on 10 dpi (Fig. 3c). The decrease in circulating lymphocytes closely followed the increase in viremia and type I/III IFN activity in serum detected in this group (Fig. 2). All group means were within established reference ranges for cattle; thus, the changes in lymphocyte quantities are indicative of relative lymphocytosis (in vaccinated animals) and relative lymphopenia (in non-vaccinated animals) after challenge.

Similar to the mean lymphocyte counts, mean monocyte counts varied by vaccination status and time after infection. Mean monocyte counts in non-vaccinated steers were not significantly different over the course of the experiment. Vaccinated steers, however, had relative monocytosis from 5 to 9 dpi (*p* <0.05) (Fig. 3d). Six individual animals exceeded the threshold for absolute monocytosis at least once, but the group means did not. The three vaccinated animals (out of 18) that lacked definitive evidence of local virus replication did not have increased monocyte counts (not shown).

Mean neutrophil counts did not differ significantly between vaccinated and non-vaccinated steers, nor did they vary significantly over the course of infection (Fig. 3b).

### Hematology: FMDV carriers vs. non-carriers

**Fig. 4:**
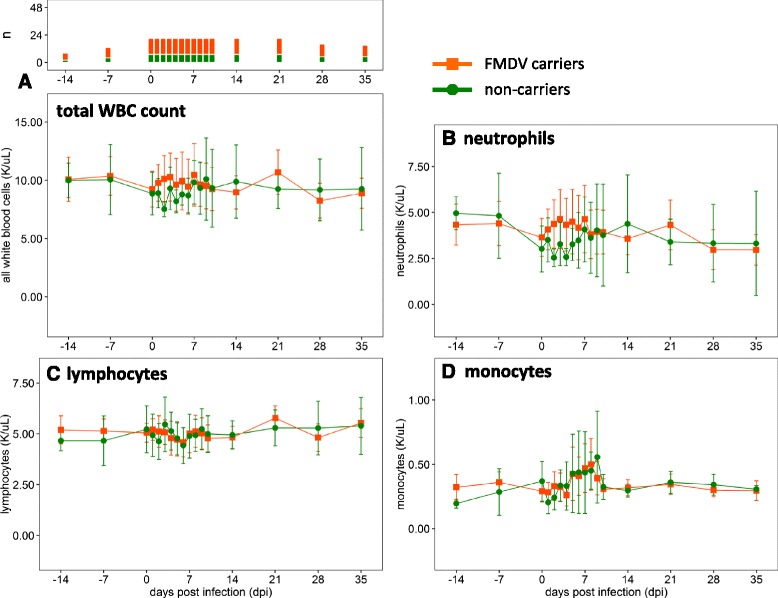
White blood cell subpopulations in carriers and non-carriers. **a** Total WBC count. **b** Neutrophils. **c** Lymphocytes. **d** Monocytes. Animals were assigned to groups based on their FMDV persistence status, without regard to their vaccination status. Persistently infected FMDV carrier animals have *orange lines* and *squares*, and non-carriers have *green lines* and *circles*. The plots show the group means with 95 % confidence intervals. The upper part of panel **a** shows the number of animals (n) that contributed data for each time point; this number is consistent for all panels. The number of animals contributing data to each time point is stable between 0 and 21 dpi

FMDV persistence status did not correlate with differences in total WBC count or any of its subpopulations. Carrier and non-carrier steers had similar mean hematological parameters over the course of infection (Fig. 4). Non-carrier steers had slightly higher mean lymphocyte counts from 3 to 10 dpi, but there was no statistically significant difference (*p* > 0.05). Overall, the observed hematological differences were more substantively associated with vaccination status than with persistence status.

In Fig. 3, animals are assigned to groups based on their FMDV vaccination status, without regard to their persistence status, whereas in Fig. 4, they are assigned to groups based on their FMDV persistence status, without regard to their vaccination status. A set of charts showing the hematology data stratified by vaccination and persistence status at the same time is included as Additional file 1: Figure S1 in the online version of this article.

### Flow cytometry: Vaccinated vs. non-vaccinated animals

Serial examinations of phenotypic characteristics of PBMCs from forty-seven steers were performed on up to 22 samples per animal by staining in duplicate and evaluating by flow cytometry. The mean coefficent of variation between replicates of the same sample was less than 3 %.

The relative size of the CD3^+^ population within the morphological lymphocyte gate (forward and side scatter) was variable over time, and trended lower in non-vaccinated animals after challenge. There was a significant difference between vaccinated and non-vaccinated animals on 4 and 5 dpi (*p* <0.05; not shown).

**Fig. 5:**
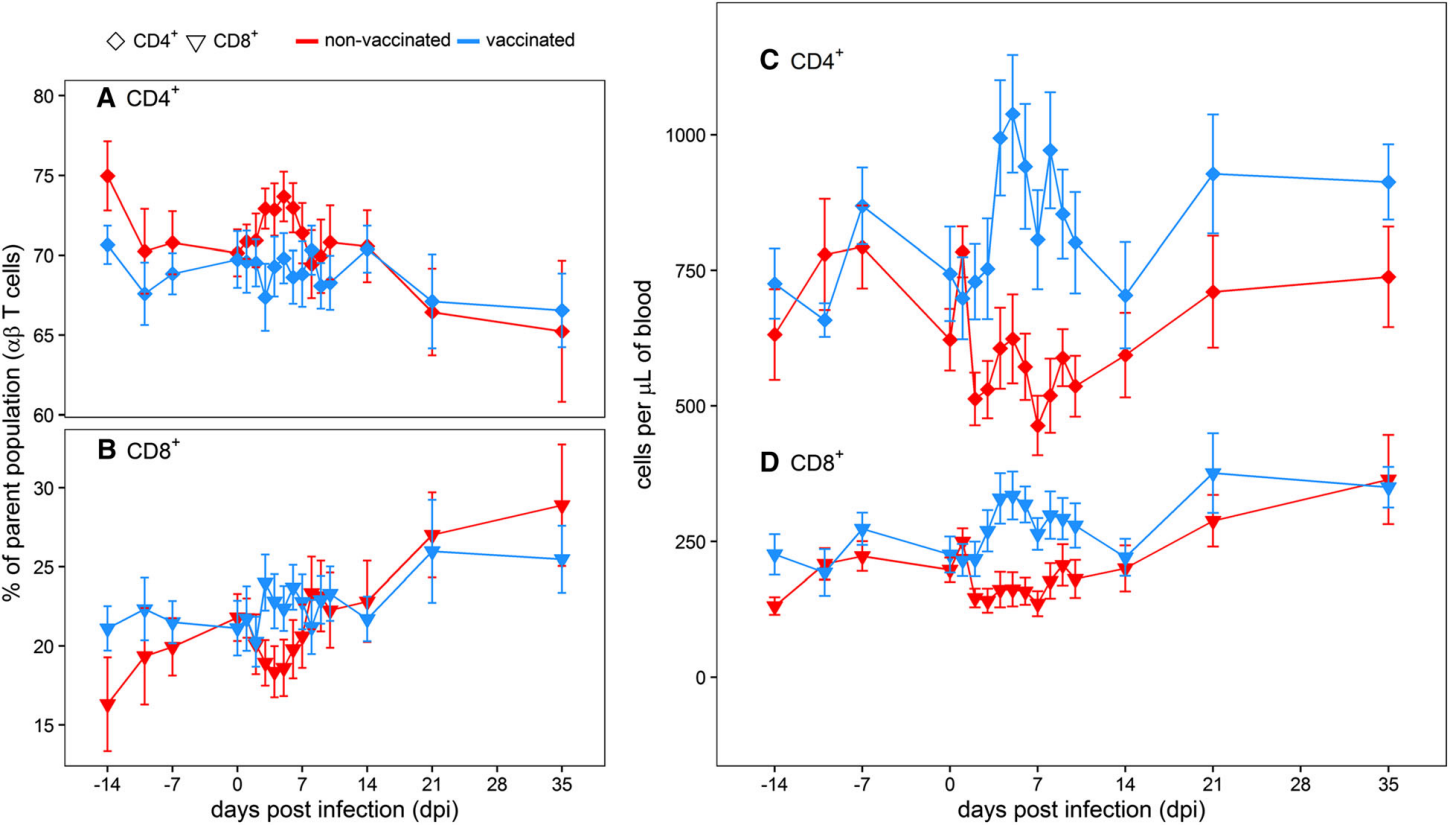
CD4^+^ and CD8^+^ αβ T cells in vaccinated and non-vaccinated animals. Panels **a** and **b** show percentage values relative to the CD3^+^ αβ T cell parent population, and panels C and D show absolute cell counts per μL of blood obtained by a dual-platform method. Group means (CD4^+^: diamonds, CD8^+^: triangles) are annotated with their 95 % confidence intervals, non-vaccinated animals are shown in *red* and vaccinated animals in *blue*. Based on relative percentage-of-parent values, there was a significant increase of CD4^+^ αβ T cells (panel **a**) and a corresponding decrease of CD8^+^ αβ T cells (**b**) in non-vaccinated animals. Translated to absolute counts, the increase in total circulating lymphocytes of up to 25 % in vaccinated animals had greater influence than the relative differences between vaccinated and non-vaccinated animals. Thus, absolute quantities of both CD4^+^ (**c**) as well as CD8^+^ αβ T cells (**d**) were significantly higher in PBMCs of vaccinated steers than in non-vaccinated steers

Based on relative percentage-of-parent values, the CD4^+^ and CD8^+^ αβ T cell subpopulations in vaccinated animals did not change significantly after challenge, but there was a significant increase of CD4^+^ αβ T cells and a corresponding decrease of CD8^+^ αβ T cells in non-vaccinated animals from 3 to 6 dpi (compared to day 0; *p* <0.05 for all 4 days, Fig. 5a, b). For each subpopulation, the difference between vaccinated and non-vaccinated animals was significant for the same time period (3 to 6 dpi, *p* <0.05). The CD4^+^/CD8^+^ ratio in non-vaccinated animals transiently increased from 3.2 at the day of challenge to 4.0 on 5 dpi, while it slightly decreased (from 3.3 to 3.1) in vaccinated animals.

In order to translate relative population measures obtained by flow cytometry to absolute counts, the percentage-of-parent values from the flow cytometry analysis were applied to the lymphocyte counts obtained with the hematology analyzer. When this conversion was performed, the increase in total circulating lymphocytes (compared to the day of challenge) of up to 25 % in vaccinated animals (Fig. 3c) had greater influence than the relative differences between vaccinated and non-vaccinated animals that have been described above. Thus, absolute quantities of both CD4^+^ as well as CD8^+^ αβ T cells were significantly higher in PBMCs of vaccinated steers than in non-vaccinated steers (*p* <0.05) from 2 to 10 dpi (Fig. 5c, d).

The absolute decrease (compared to the day of challenge) of the total lymphocyte count in non-vaccinated animals also influenced the counts of the CD4^+^ and CD8^+^ αβ T cell subpopulations. In relative terms, CD4^+^ αβ T cells had been transiently increased in non-vaccinated animals, while CD8^+^ αβ T cells had shown a complementary decrease (Fig. 5a, b). The relative increase in CD4^+^ cells partially counteracted the total decrease in lymphocytes, whereas the decrease in CD8^+^ cells became more pronounced once absolute counts were taken into account (Fig. 5c, d). Absolute CD4^+^ cell counts during acute infection fluctuated substantially more than CD8^+^ counts (Fig. 5c, d). On day 5 after infection, the day with the largest CD4^+^/CD8^+^ ratio (4.0, see above) as well as the nadir of the lymphocyte count in non-vaccinated animals (Fig. 3c), the absolute CD8^+^ cell count in non-vaccinated animals was reduced by approximately 20 % compared to day 0 (166 ± 34 vs. 202 ± 30 per μL of blood; means and CI95, *p* = 0.03), while the absolute CD4^+^ count was similar to the initial level (624 ± 85 vs. 619 ± 66, *p* = 0.91).

### Flow cytometry: FMDV carriers vs. non-carriers

There were no significant differences between carrier and non-carrier animals in the kinetics of any T cell population investigated herein, independent of whether vaccinated and non-vaccinated animals were analyzed together or separately, and independent of the measurement scale (relative or absolute).

### FMDV-specific humoral immune response

**Fig. 6:**
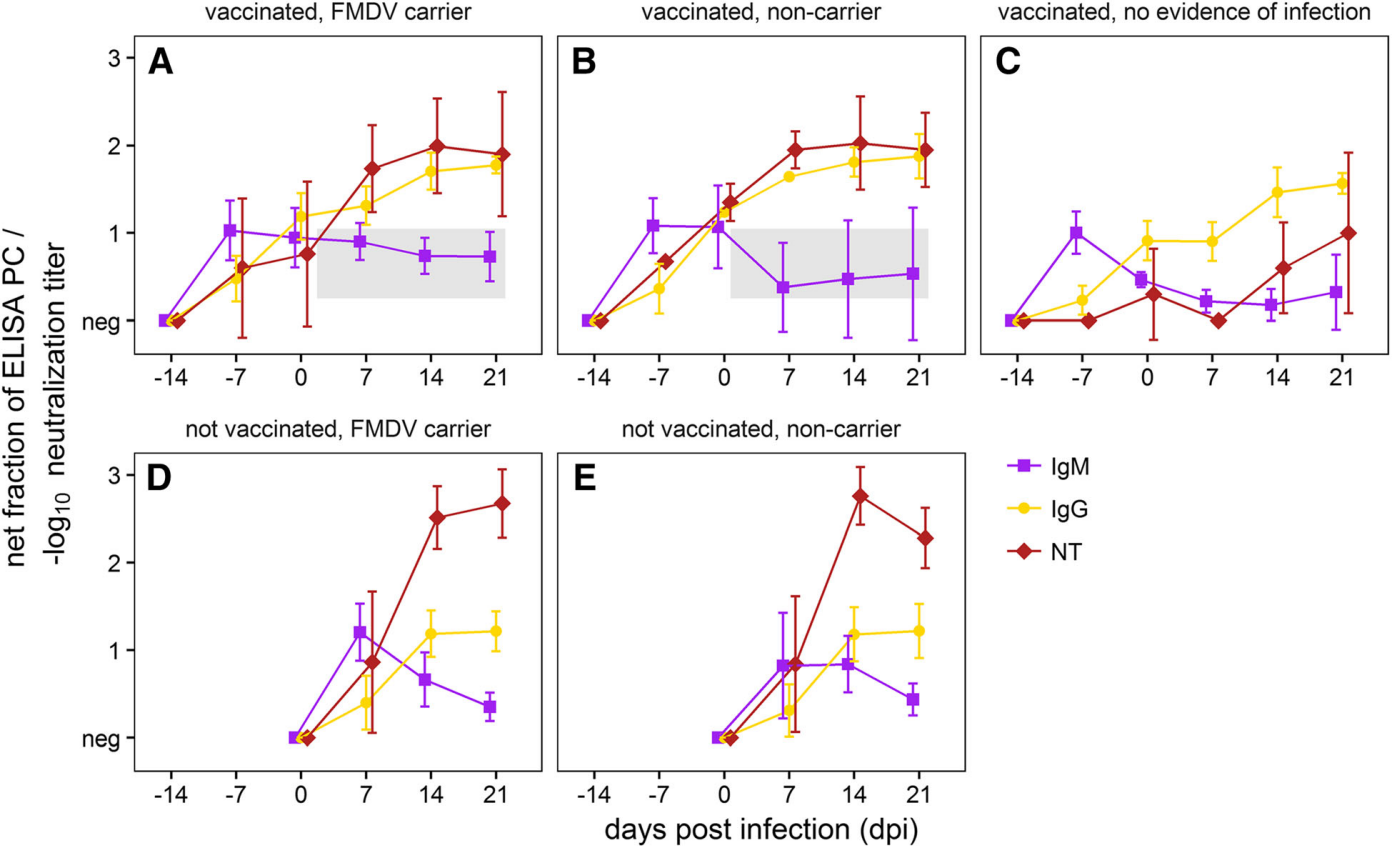
Serological response to FMDV vaccination and challenge. Animals are grouped by vaccination status (“vaccinated”, “not vaccinated”) and FMDV persistence status (“carrier”, “non-carrier”, or “no evidence of infection” for vaccinated non-carriers without virological evidence of FMDV replication after challenge). Each panel shows group means with standard deviation for one combination of the two categories. In all five panels, *purple lines with square* markers show FMDV-specific IgM levels, *golden lines with round markers* show FMDV-specific IgG and *maroon lines with diamond markers* show FMDV A24 neutralization titers. The *gray boxes* highlight the potentially different post-challenge IgM kinetics in vaccinated carriers and non-carriers (see text). PC = positive control

In vaccinated animals, no FMDV-specific antibodies were detected at 4 dpv (by ELISA only; the 4-dpv sample was not tested in the SNT), but all vaccinated animals had developed FMDV-specific IgM and IgG by 7 dpv, and most had detectable neutralizing antibody at the day of challenge (14 dpv; Fig. 6).

All vaccinated animals had detectable FMDV-specific IgM on the day of challenge. Vaccinated animals that went on to become FMDV carriers maintained their pre-challenge FMDV IgM levels for the duration of the study, whereas FMDV-specific IgM in vaccinated animals that did not become persistently infected was markedly reduced by 7 dpi (see boxes in Fig. 6a, b). In contrast, FMDV-specific IgM in serum from non-vaccinated animals was first detected on day 5 after challenge. Levels increased quickly over the next few days and peaked on 9 dpi. In carriers and non-carriers IgM then declined steadily until the end of the experiment, but remained detectable throughout.

After the first detection on 7 dpv, FMDV-specific IgG in vaccinated animals increased steadily until 14 dpi. All vaccinated animals had detectable FMDV-specific IgG on the day of challenge. In non-vaccinated animals, specific IgG was first detectable on 6 dpi and rose quickly until 14 dpi. After 14 dpi, the levels of specific IgG remained stable in vaccinated and non-vaccinated animals, but the final levels in vaccinated animals were higher than in non-vaccinated animals (Fig. 6a, b).

Neutralizing antibody levels in vaccinated and non-vaccinated animals generally correlated with FMDV-specific IgG rather than with IgM. However, in contrast to the ELISA data, final levels of neutralizing antibodies were higher in animals that had not been vaccinated before challenge and thus had developed fulminant FMD (Fig. 6d, e). The three vaccinated animals without virological evidence of local FMDV replication or dissemination had lower specific antibody levels than other vaccinated animals, both before and after challenge (Fig. 6c); however they did have increases in IgG and neutralization titers after challenge.

Apart from the slightly different IgM kinetics in vaccinated animals after challenge, there was no difference in FMDV-specific circulating antibody between carriers and non-carriers.

### Isotype-specific characterization of anti-FMDV B cells

**Fig. 7:**
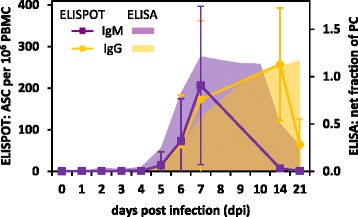
FMDV-specific humoral immune response in 6 non-vaccinated animals. Antibody-secreting cells (ASC) in peripheral blood were counted with ELISPOT assays, and circulating antibody was measured by sandwich ELISAs. IgM is shown in *purple*, IgG in *gold*. Error bars show sample standard deviation. PC = positive control

FMDV-specific circulating B cells of six non-vaccinated animals were evaluated using an antibody ELISPOT assay. Anti-FMDV antibody-secreting cells were first detected in non-vaccinated animals on 5 dpi. IgM-secreting cells peaked between 7 and 14 dpi, and had disappeared from peripheral blood by 14 dpi. IgG-secreting cell counts in circulation increased until 14 dpi, and then declined. Low levels of FMDV IgG-secreting cells remained detectable in peripheral blood until the end of the experiment (Fig. 7).

## Discussion

The present study examined the systemic response to FMDV infection in cohorts of non-vaccinated and vaccinated cattle with the purpose of investigating systemic immune system factors that may correlate with the divergence between FMDV carriers and non-carriers. Persistent infection can follow either a clinical or a subclinical FMDV infection and generally occurs at the same rate regardless of vaccination reviewed in [67]. The protective efficacy of Ad5-FMDV vaccination against disseminated disease has been demonstrated previously [46, 68, 69], but the incidence and characteristics of persistent infection after challenge of Ad5-FMDV-vaccinated cattle was studied here for the first time. Complete blood counts, T lymphocyte subpopulations, type I/III IFN response, as well as FMDV-specific antibody production and activity were compared between vaccinated and non-vaccinated animals and between carriers and non-carriers at an unprecedented level of detail. Before challenge, vaccinated cattle had detectable humoral immunity against FMDV. After challenge, all non-vaccinated steers became viremic and developed clinical disease, while the vaccinated steers did not. This divergence coincided with distinctive differences in several parameters. On the other hand, only slight differences were identified between animals that went on to become FMDV carriers compared to those which cleared the infection.

The present study confirmed earlier findings that summary WBC levels in non-vaccinated animals remain within the clinical reference range during acute FMDV infection and do not change significantly from the outset [28]. There was, however, a significant change in lymphocyte levels, which transiently decreased in non-vaccinated animals after FMDV challenge. This relative lymphopenia was significant compared to pre-infection levels; however, the animals were never lymphopenic relative to the established clinical reference range [56], which underscores the utility of reference change values over population-level standards in animal research [70].

Lymphopenia is a common feature of acute viral infections [71]. It is a well-described phenomenon in FMDV-infected pigs [24, 25, 72], and has previously been reported in FMDV-infected cattle in at least two studies [20, 26]. There are several mechanisms by which viruses can cause lymphopenia. Lymphocytes may be redistributed from the blood into infected tissues, e.g. through the action of type I IFNs. Alternatively, apoptosis of lymphocytes can be a direct result of viral infection or caused indirectly through cytokine induction [73]. In the present study, the decrease in circulating lymphocytes closely followed the increase in systemic type I/III IFN activity, which in turn was highly correlated with FMDV viremia. Thus, these findings are consistent with the observed lymphopenia being due to IFN-induced lymphocyte migration from the blood to sites of infection or lymphoid tissues [74]. The CBC only examines circulating cells; animals with a relative lymphopenia may have normal or even increased levels of total body lymphocytes and the reduction in circulating lymphocytes alone does not necessarily represent a decline in immune competence. It has been argued that transient virus-induced and IFN-mediated lymphopenia is, in fact, a physiological response and beneficial to the host reviewed in [74].

Similarly, increased lymphocyte counts, as were detected in the vaccinated animals in the present study, are often seen after antigenic stimulation [71]. The recall response after challenge of vaccinated animals is the result of an increased quantity of primed antigen-specific B and T cells, which can expand more quickly. Memory cells are activated more readily than naïve cells and can respond to lower doses of antigen [75].

As the bridge between innate and adaptive immunity, monocytes and macrophages are a key component in antigen presentation, phagocytosis, and viral clearance [76]. They usually form only a small part of the circulating leukocyte pool. In the present study, monocyte numbers did not change significantly after vaccination, or after FMDV infection of non-vaccinated animals, but there was a striking pattern in vaccinated steers after challenge. Four days after challenge, and two days after circulating lymphocyte counts had started to increase, monocyte counts rose sharply and remained high (approximately 50 % above baseline) for several days. Sigal et al. [77] have previously reported a similar increase of monocytes in FMDV-vaccinated animals 1 week after challenge.

Monocytes are released into circulation from the bone marrow and spleen in response to inflammation signals [78], and move quickly to sites of infection where they differentiate into macrophages or dendritic cells. Increased antigen presentation and lymphocyte activation stimulate additional monocyte recruitment through positive feedback loops [79]. Opsonization by specific antibody and phagocytosis are major factors in the immune defense against FMDV [80, 81]. Additionally, pDC activation by FMDV is also substantially enhanced in the presence of FMDV-specific antibodies [82]. In the vaccinated animals, protection from FMD occurred in the presence of abundant anti-FMDV immunoglobulin combined with elevated circulating monocyte counts, both of which were likely contributors to the effective immune response.

It has previously been shown that there is a strong systemic type I/III IFN response during acute FMDV infection of cattle [19, 21, 22], and this was again confirmed in the large cohort of unvaccinated cattle in the present study. High IFN activity is also detectable in tissues of FMDV-infected animals, but it is restricted to sites of virus replication [22]. It is unclear how much of the locally produced IFN enters the vasculature, and it has been suggested that the circulating type I/III IFN is instead produced by CD4^+^ pDCs interacting with immune-complexed virus [18]. A flow cytometric analysis of ex vivo interferon production in pDCs from the FMDV-infected animals would have provided useful information in this regard, but this was beyond the scope of the present study.

No infectious virus or FMDV RNA was detected in serum of vaccinated animals in the present study; thus, the lack of a systemic type I/III IFN response could be explained by the absence of the crucial stimulus for its production. However, induction of transcription of IFN genes and IFN bioactivity was detected in pharyngeal tissues of vaccinated cattle from this study [38].

In addition to its critical role in innate immunity, type I IFN can act directly on both CD4^+^ and CD8^+^ T cells, positively or negatively influencing their function reviewed in [83]. T cells are the dominant lymphocyte population in peripheral blood of cattle [84]. For the first time, the present study identified significant changes in αβ T cells in non-vaccinated steers challenged with FMDV, complementing earlier work on γδ T cells [85]. After infection, non-vaccinated animals had a significant decrease in the relative quantity of CD8^+^ αβ T cells for several days, while the same cell population in vaccinated animals remained stable. Conversely, the relative CD4^+^ αβ T cell population in non-vaccinated animals was larger during that time, but did not change in vaccinated animals. In order to determine which subset was responsible for the change in the CD4^+^/CD8^+^ ratio, lymphocyte counts from the CBC were applied to the relative changes of lymphocyte subpopulations – commonly referred to as the dual-platform method of obtaining absolute counts [60]. The multicolor staining panel used in the present study also accounts for non-T lymphocytes and γδ T cells. Using this method, absolute CD4^+^ counts in non-vaccinated animals were found to be stable during acute infection, while the absolute CD8^+^ counts were significantly decreased. This indicates selective depletion (or redistribution) of circulating CD8^+^ αβ T lymphocytes in FMDV-infected non-vaccinated cattle. A similar pattern has been previously described in swine [24, 25], compounded by evidence of functional impairment (e.g., mitogen unresponsiveness) of the residual circulating T cell population. A variety of explanations for these observations have been suggested [74], including FMDV infection of T lymphocytes or T-cell suppression by IL-10 overproduction [86]. Joshi et al. [87] reported a similarly inhibited response to mitogen in FMDV-infected bovine lymphocytes, but other studies found no evidence of changes in CD4^+^ and CD8^+^ populations, T-cell impairment or increased circulating IL-10 during acute FMDV infection in small cohorts of cattle [27, 28].

The time course of the FMDV-specific antibody response observed in the present study was similar to previously published results from other groups [27, 88–90]. Direct comparisons of the magnitude of the response, however, are impossible because of differences in the test format (endpoint dilution vs. optical density at a fixed dilution, use of monoclonal anti-isotype vs. polyclonal anti-isotype antibodies, etc.). In addition, neither system allows for a fully quantitative comparison of different immunoglobulin isotypes within a sample, only of relative levels of the same isotype between samples [89].

When comparing the antibody development after challenge between FMDV-vaccinated and non-vaccinated animals, Mulcahy et al. [89] saw a transient increase of specific IgM in non-vaccinated animals, followed by a sustained increase of specific IgG. This is the classical pattern of a primary immune response [91] that was also seen in non-vaccinated animals in the present study. Similar to what has been discussed for cellular adaptive responses, secondary or recall antibody responses differ from primary responses. They occur more rapidly, they consist of relatively more IgG than IgM, and they are of higher affinity [75]. Indeed, no rise in specific IgM was seen after challenge in the vaccinated animals in the present study, but FMDV-specific IgG levels in vaccinated animals did increase after challenge.

This increase was also seen in the three vaccinated animals that lacked evidence of local virus replication, possibly because antibody-bound FMDV was recognized by the immune system and induced an anamnestic response. Alternatively, there could have been low-level replication that was not directly detectable and which was insufficient to induce measurable antibodies against non-structural proteins. Overall, the data support these animals not having been infected. Strikingly, they only had low neutralizing antibody titers and no detectable type I/III interferon in serum before challenge; thus, the specific mechanisms that facilitated their apparent sterile protection remains undetermined. While the neutralizing antibody profiles between serum and nasal fluid of FMDV-vaccinated pigs are similar, this is not necessarily true for cattle [92], and the low serum titers are not indicative of a similarly low mucosal response.

Persisting serum IgM is used to diagnose chronic viral infections such as hepatitis B and C in humans. Contrary to the results presented here, Salt et al. [90] had not seen such a pattern for FMDV-specific IgM in carriers. In the present study, modestly elevated FMDV-specific IgM was detected in carriers. This may be because persistent FMDV infection is not highly productive, unlike the aforementioned chronic infections. It is interesting to note, too, that while IgM remains detectable, IgM-secreting cells disappear from circulation, possibly indicating a more localized production of the antibodies-similar to what has been described for the IgA response to persistent infection [42]. The magnitude of the difference in IgM kinetics between carriers and non-carriers, however, appears to be too small to be of diagnostic utility. As has been reported previously reviewed in [4], no component of the serum antibody response in the present study was significantly different between carriers and non-carriers.

## Conclusions

This study corroborates the notion that vaccination and prevention of clinical disease offer no protection against either primary or persistent FMDV infection [12, 38, 90]. This is the first use of high-resolution hematology and multi-color flow cytometry data from a large cohort of animals, all exposed to the virus under simulated-natural conditions and closely monitored for the full course of infection. Lymphopenia and selective CTL depletion were found to be significant phenomena during the acute infection of naïve cattle, in contrast with a robust cellular immune response in vaccinated animals. However, none of the systemic parameters examined–type I/III interferon, FMDV-specific antibodies, circulating leukocyte populations and T-lymphocyte subsets–were associated with the FMDV carrier state divergence, emphasizing the highly localized nature of persistent FMDV infection. This suggests that tissue-level studies of sites of persistence may be required to elucidate the mechanisms associated with establishment and resolution of persistent FMD, and such studies are currently underway in our laboratory.

## Additional files

Additional file 1: Figure S1. White blood cell subpopulations by vaccination and persistence status. A: Total WBC count. B: Neutrophils. C: Lymphocytes. D: Monocytes. Animals were assigned to one of four groups based on their FMDV vaccination and persistence status. Group means are shown with 95 % confidence intervals. Means are marked with orange circles for carrier animals and green circles for non-carriers; individual time points are connected with blue lines for vaccinated animals and with red lines for non-vaccinated animals. Small numbers at the bottom of each panel state the number of animals that contributed data to each time point. (PDF 595 kb) Additional file 2: Table S1. Full dataset in tab-separated format. (TSV 70 kb)

## Abbreviations

ANOVA: Analysis of variance
ARS: Agricultural Research Service
BB: Blocking buffer
CAT: Chloramphenicol acetyltransferase
CBC: Complete blood count
CD: Cluster of differentiation
CI95: 95 % confidence interval
CO_2_: Carbon dioxide
C_t_: Cycle threshold
CTL: Cytotoxic T lymphocyte (s)
Dpi: Days post-infection
Dpv: Days post-vaccination
ELISA: Enzyme-linked immunosorbent assay
ELISPOT: Enzyme-linked immunospot
FACS: Fluorescence-activated cell sorting
FADRU: Foreign Animal Disease Research Unit
FBS: Fetal bovine serum
FMD: Foot-and-mouth disease
FMDV: Foot-and-mouth disease virus
GCN: Genome copy number
HEPES: 4-(2-hydroxyethyl)-1-piperazineethanesulfonic acid
HRP: Horseradish peroxidase
IFN: Interferon
IgG: Immunoglobulin gamma
IgM: Immunoglobulin mu
IL-10: Interleukin 10
INP: Intranasopharyngeal
K_2_EDTA: Dipotassium ethylenediaminetetraacetic acid
LFBK: Lois’ and Fran’s bovine kidney cells
MEM: Minimum essential medium
Mx: Mx1, Myxovirus resistance protein 1
nFPC: Fraction of the net OD of the positive control
NSP: Non-structural protein
OD: Optical density
OPF: Oropharyngeal fluid (s)
ORISE: Oak Ridge Institute for Science and Education
PBMC: Peripheral-blood mononuclear cell (s)
PBS: Phosphate-buffered saline
PBST: Phosphate-buffered saline with TWEEN (R)
PC: Positive control
pDC: Plasmacytoid dendritic cell
PIADC: Plum Island Animal Disease Center
RNA: Ribonucleic acid
RPMI: Roswell Park Memorial Institute
RT-PCR: Reverse transcription polymerase chain reaction
SNT: serum neutralization test
TCID_50_: 50 % tissue culture infective doses
TTE: 1,1,2-trichlorofluoroethane
USDA: United States Department of Agriculture
WBC: White blood cell (s)
