# Supplementary figures and images for: Systemic immune response and virus persistence after foot-and-mouth disease virus infection of naïve cattle and cattle vaccinated with a homologous adenovirus-vectored vaccine
Source: BMC Vet Res. 2016 Sep 15;12:205. doi: 10.1186/s12917-016-0838-x (PMC5025598; doi:10.1186/s12917-016-0838-x)

## A: all white blood cells

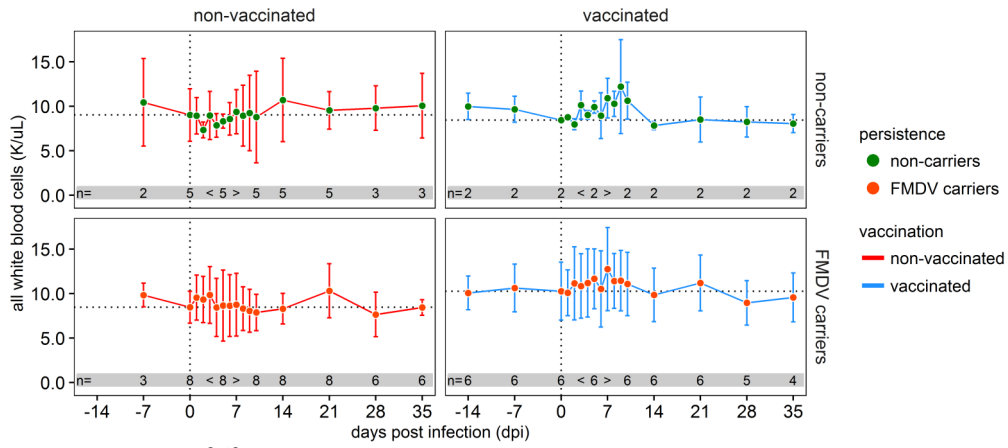

## B: neutrophils

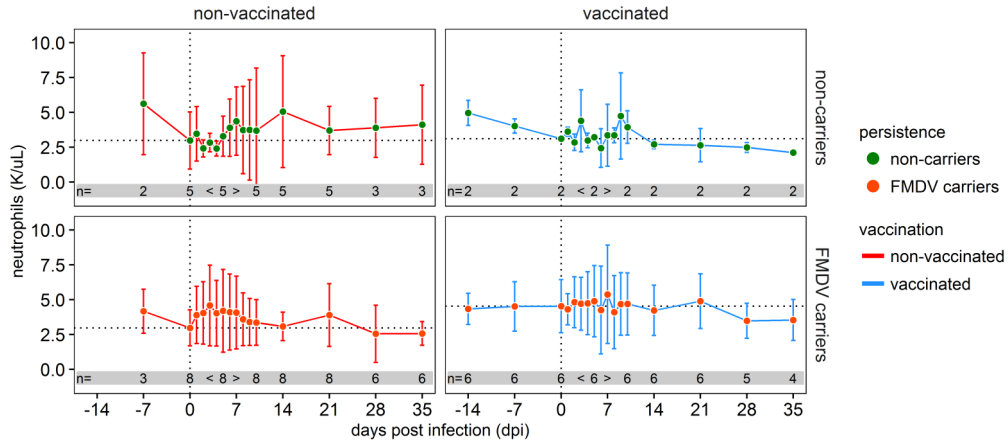

## C: lymphocytes

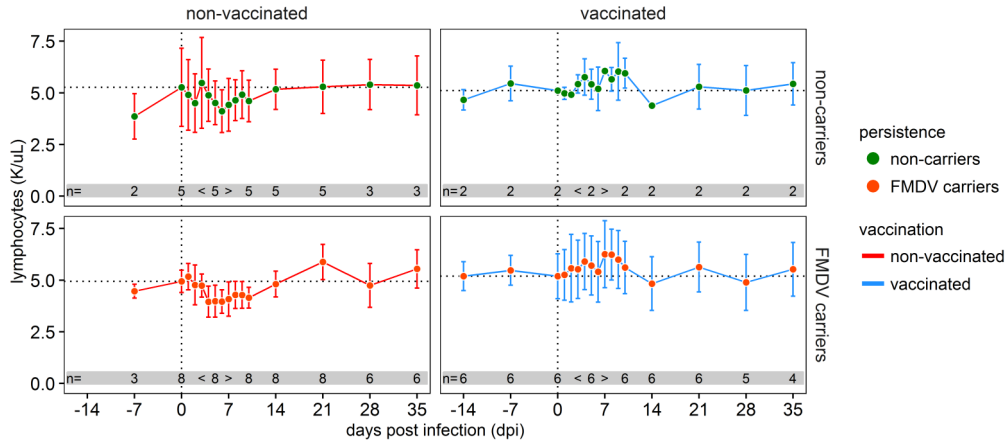

## D: monocytes

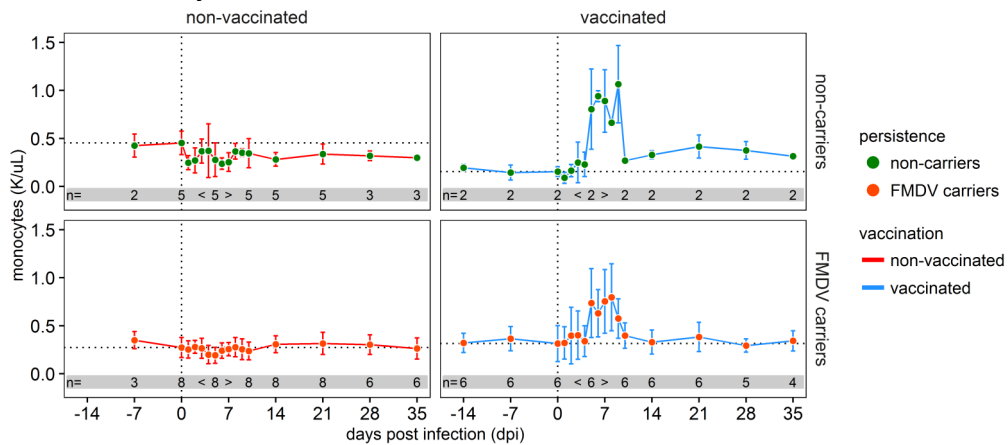

Supplement: Additional file 1: Figure S1. — White blood cell subpopulations by vaccination and persistence status. A: Total WBC count. B: Neutrophils. C: Lymphocytes. D: Monocytes. Animals were assigned to one of four groups based on their FMDV vaccination and persistence status. Group means are shown with 95 % confidence intervals. Means are marked with orange circles for carrier animals and green circles for non-carriers; individual time points are connected with blue lines for vaccinated animals and with red lines for non-vaccinated animals. Small numbers at the bottom of each panel state the number of animals that contributed data to each time point. (PDF 595 kb) [file 12917_2016_838_MOESM1_ESM.pdf]
